# Antibody-mediated disruption of the SARS-CoV-2 spike glycoprotein

**DOI:** 10.1038/s41467-020-19146-5

**Published:** 2020-10-21

**Authors:** Antoni G. Wrobel, Donald J. Benton, Saira Hussain, Ruth Harvey, Stephen R. Martin, Chloë Roustan, Peter B. Rosenthal, John J. Skehel, Steven J. Gamblin

**Affiliations:** 1 Structural Biology of Disease Processes Laboratory, Francis Crick Institute, NW1 1AT London, UK; 2 Worldwide Influenza Centre, Francis Crick Institute, NW1 1AT London, UK; 3 Structural Biology Science Technology Platform, Francis Crick Institute, NW1 1AT London, UK; 4 Structural Biology of Cells and Viruses Laboratory, Francis Crick Institute, NW1 1AT London, UK

**Keywords:** SARS-CoV-2, Cryoelectron microscopy

## Abstract

The CR3022 antibody, selected from a group of SARS-CoV monoclonal antibodies for its ability to cross-react with SARS-CoV-2, has been examined for its ability to bind to the ectodomain of the SARS-CoV-2 spike glycoprotein. Using cryo-electron microscopy we show that antibody binding requires rearrangements in the S1 domain that result in dissociation of the spike.

## Abstract (web summary)

Here, the authors characterise the binding and neutralisation properties of CR3022, a neutralising Ab isolated from a convalescent SARS patient, against SARS-CoV-2 spike trimers and show using Cryo-EM the disruption of SARS-CoV-2 spike by CR3022 Fab.

## Introduction

The spike (S) membrane glycoproteins of severe acute respiratory syndrome coronavirus 2 (SARS-CoV-2), like those of other coronaviruses, are responsible for receptor binding and membrane fusion at the beginning of virus replication^1–4^. They are also the antigens recognised by antibodies that neutralise virus infectivity. Because of the urgency of the pandemic and the similarities between SARS-CoV and SARS-CoV-2^5,6^, monoclonal antibodies prepared from convalescent patients at the time of the SARS CoV-1 outbreak^7^ have been tested for their reactivities with SARS-CoV-2 S. Among them CR3022 was found to target the receptor-binding domain (RBD) of the spike^8^, which, together with the N-terminal domain (NTD), forms the main antigenic surface of the S1 domain. S from SARS-CoV and SARS-CoV-2 share about 77% amino acid identity and bind the same receptor, ACE2^5^. A recent crystal structure^9^ of the complex of the SARS-CoV-2 RBD and the Fab of CR3022 indicates that the antibody binds to a conserved region of the RBD, which does not overlap with the receptor-binding surface. Trimeric S on the virus surface is thought to adopt two main conformations: closed, in which much of the RBD surface is buried inside the trimer and unable to bind the receptor ACE2, and open, in which one RBD protrudes from the trimer and can bind the receptor^10,11^. Neither of these conformations is able to accommodate the binding of CR3022 although it has been hypothesised that an S conformation with two-RBDs-up potentially could^9^.

To investigate how CR3022 interacts with intact S of SARS-CoV-2 we use binding and neutralisation assays and study the structure of the complex by electron cryo-microscopy. Our data indicate that CR3022 binding accompanies dissociation of the S trimer. We also find that CR3022 does not neutralise SARS-CoV-2.

## Results

### Binding of CR3022 Fab to SARS-CoV-2 spike

We expressed CR3022 Fab and the stabilised, trimeric ectodomain of the SARS-CoV-2 S in mammalian cells and performed biolayer interferometry to investigate Fab binding to immobilised trimers. The protein was obtained by the same protocol detailed in our recent study^12^ that describes a 2.6 Å structure of the closed pre-fusion conformation of the trimeric SARS-CoV-2 S (Supplementary Fig. 1a, b). Analysis of CR3022 binding provides a dissociation constant (*K*_d_) of 80 nM towards the trimeric spike (Supplementary Fig. 1c). This value is similar to those reported before in experiments measuring Fab binding to isolated RBD^8,9^.

### Structure of CR3022 Fab bound to SARS-CoV-2 spike

**Fig. 1:**
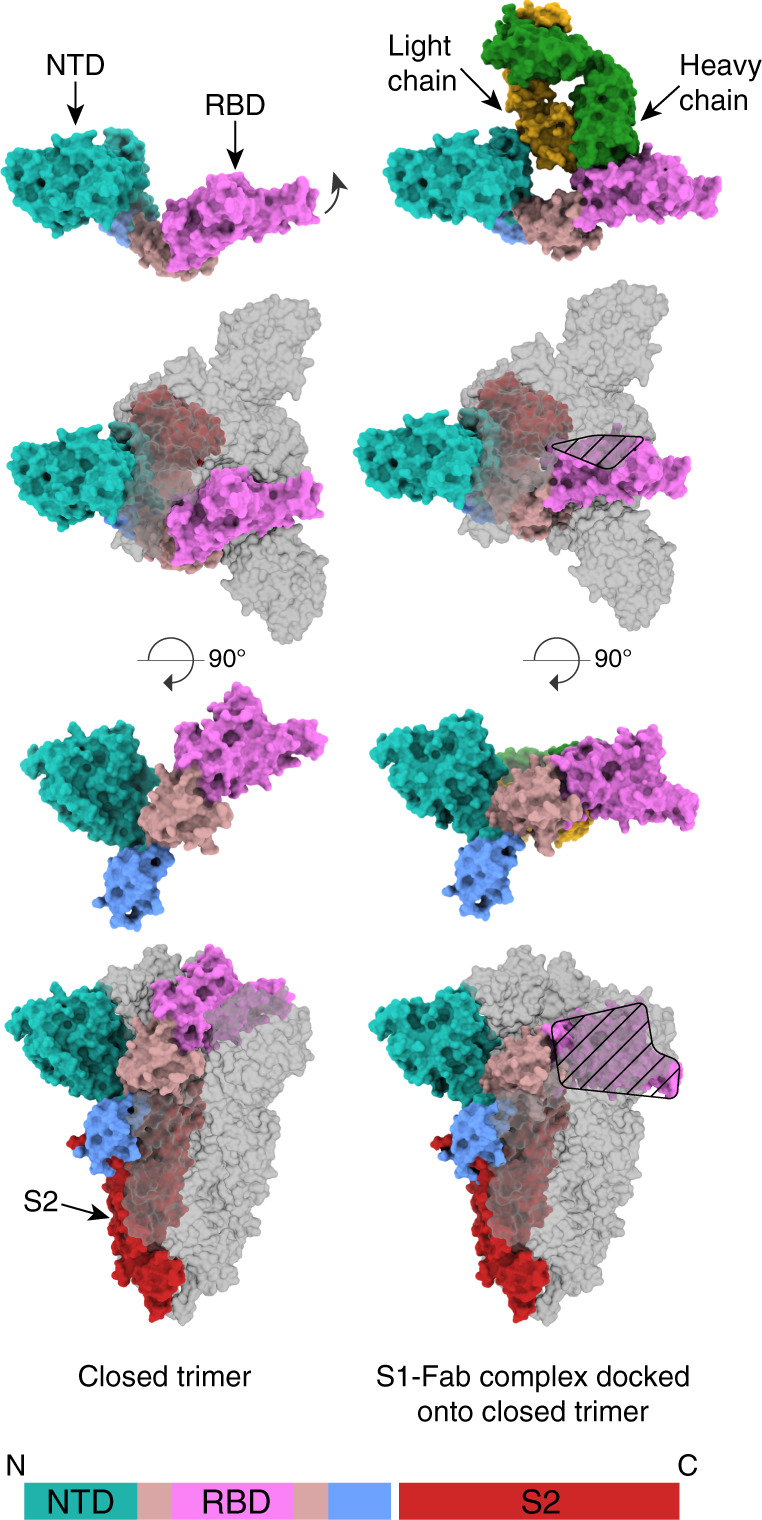
Structure of CR3022 Fab bound to S in space-filling representation. Column on the left shows the native S, while that on the right shows S with bound CR3022. The top row shows a monomer of S1 without and with CR3022. The individual subunits are labelled and coloured according to the bar diagram at the bottom of the figure. The second row shows a full S1/S2 trimer in the same orientation as the top panel with one monomer coloured according to the top panel but the other S1 subunits are in grey. Where part of a coloured subunit is eclipsed by a grey subunit the surface is mottled; where there is a steric clash the surface of the coloured subunit is hatched. The third and fourth rows are orthogonal views to the top two rows with the trimer threefold axis vertical. The S2 chain associated with the coloured S1 chain is shown in red.

We used cryo-EM to determine the molecular basis of S recognition by CR3022 (Fig. 1). The single-particle reconstructions generated a map with a global resolution of 3.7 Å resolution (Supplementary Fig. 2 and Supplementary Table 1) and enabled the description of all the domains of S1 and the unbiased building of the Fab and RBD. The high resolution of the data is partly accounted for by the formation, over time, of an adventitious but stable dimer of two CR3022/S1 complexes (Supplementary Fig. 2). The reconstruction shows that the CR3022 Fab binds an epitope on the RBD that is not accessible in either the open or closed forms of trimeric S. These data are consistent with the results from the crystal structure of CR3022 Fab bound to the spike RBD^9^. Binding is made possible by the dissociation of the trimeric S into monomers presumably driven by the binding energy of the Fab. Dissociation also results in the S2 domain having no interaction surface with S1 so the two remain covalently tethered but rotationally independent of each other. The S2 domain is not visualised in the reconstruction.

The structure of the S1 domain, to which the Fab is bound, has undergone significant rearrangements (Fig. 1). The RBD has rotated by about 30 degrees, the adjacent intermediate domain by somewhat less and the entire protein has extended to accommodate the Fab (Fig. 1). The overall interaction area between the Fab and RBD is 930 Å^2^, of which 590 and 340 Å^2^ are contributed by the interaction between the CDRs of the heavy and light chains respectively. A second, smaller interface occurs between the V_L_ chain of the Fab with the NTD and accounts for an additional 270 Å^2^ of interaction surface.

**Fig. 2:**
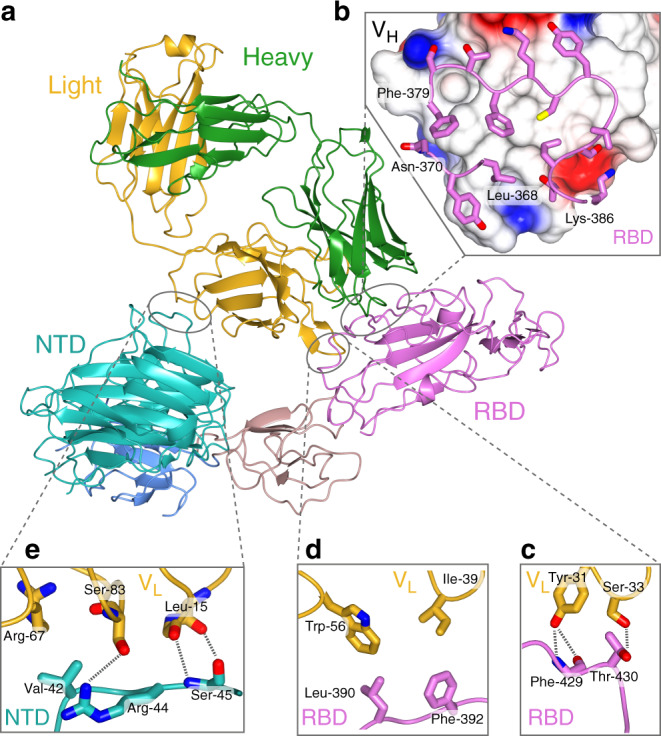
Interactions between CR3022 Fab and SARS-CoV-2 spike protein. **a** Ribbon representation of the interactions between CR3022 Fab (with light chain in yellow and heavy chain in green) and the S1 domain of SARS-CoV-2 spike (coloured according to Fig. 1). **b** Zoom view of the interaction between the Fab heavy chain, shown as a surface representation coloured according to surface potential, and a worm-and-side-chain representation of two stretches of RBD (residues 379–386 and 368–370) shown in pink. There is a notable hydrophobic component in the centre of the interaction with a number of electrostatic interactions on the outside. Interactions between CR3022 light chain (yellow) with RBD (**c**, **d**) and NTD (**e**).

The most substantive component of the interface between CR3022 and the RBD involves the heavy chain of the Fab interacting with one short loop of the RBD (residues 368:370) and a second longer loop (residues 374:386) (Fig. 2a, b). The interface of these two loops presents a strongly hydrophobic interaction to the Fab flanked (top and bottom in Fig. 2b) by electrostatic interactions. The light chain of the Fab makes two smaller loop interactions; residues Phe-429 and Thr-430 of the RBD make hydrogen bond interactions (Fig. 2c) while Ile-39 and Trp-56 make hydrophobic interactions (Fig. 2d). Finally, a loop on the NTD (residues 42:45) makes a series of potential hydrogen bond interactions with the V_L_ chain of CR3022 as well as a hydrophobic contact between Val-44 of the NTD with the aliphatic moiety of the side-chain of an arginine from the Fab (Fig. 2e).

There are two reasons for Fab binding requiring the breakdown of the S trimer. First, the conformation in which S1 binds to the Fab cannot be accommodated in either the closed or open forms of the trimer. Second, the way the Fab binds to the RBD/NTD would generate considerable steric clashes within the trimeric S (Fig. 1). The most notable clashes within the trimer occur between the CR3022-bound S1 and the RBD of one neighbouring monomer and the NTD and intermediate subdomains of the second neighbouring monomer. This indicates that the S1 conformation observed in the present structure is not compatible with any of the possible pre-fusion conformations of the spike, even with one, two, or three RBDs erect. Moreover, the dimerisation of the S–Fab complex, although the biological relevance of it is unclear, would further accentuate this steric incompatibility.

To look at the timescale of CR3022–S1 complex formation we collected datasets at different times after mixing: 60 s, 5 min, and 48 h. There was no evidence in any of the samples of trimeric S bound to Fab (Supplementary Fig. 2), suggesting that the binding event rapidly captures a transient, probably poorly populated, species of S that is able to bind Fab, resulting in the disruption of the SARS-CoV-2 S trimer. Whether the trimer is permanently dissociated with this S construct, which does not have a basic cleavage site between the S1 and S2 chains, is not accessible from either our structural or biophysical studies. However, biologically, when S cleavage has resulted in the formation of S1 and S2 chains, CR3022 binding would be expected to lead at least to release of S1 as an S1–Fab complex, from membrane-associated S2.

### Neutralisation of SARS-CoV-2 by CR3022 Fab

To further characterise CR3022, we carried out plaque reduction neutralisation assays. Even though the experimental design compensates for the substantially weaker binding of CR3022 Fab to SARS-CoV-2 than to SARS-CoV, in terms of fraction bound, our data show no sign that CR3022 neutralises SARS-CoV-2 (Supplementary Fig. 3). While this result is in agreement with data reported previously for CR3022 binding to SARS-CoV-2^9^ it is in contrast to data with SARS-CoV and perhaps surprising given the molecular mechanism described here. We suggest that a potential explanation for these observations relates to the kinetics of the binding of CR3022 to the two different viruses. Binding of CR3022 to the SARS-CoV spike is strengthened by comparison to SARS-CoV-2 likely because of differences in the epitope sequence such as substitution of Thr-359 by Ala-372 and of Ala-372 by Pro-384 in SARS-CoV and SARS-CoV-2, respectively. Mechanistically the “off” rate for SARS-CoV-2 suggests a short half-life for the S-CR3022 Fab complex that could account for the failure of the Fab to neutralise the virus.

## Discussion

Overall, our findings agree with those presented by Yuan et al.^9^, who reported a similar affinity of CR3022 Fab for the RBD, mapped the SARS-CoV-2 RBD complex structurally, and also reported that CR3022 does not neutralise SARS-CoV-2. The structure of the CR3022 Fab in complex with SARS-CoV-2 S we present suggests CR3022 binds the spike as it samples a conformational space, a phenomenon perhaps not dissimilar to that recently reported for hidden epitopes on influenza haemagglutinin^13,14^. However, upon binding CR3022, the S1 domain of the SARS-CoV-2 spike assumes a new conformation incompatible with the trimeric conformation of the pre-fusion spike.

## Methods

### Construct design

A construct coding for the SARS-CoV-2 spike glycoprotein ectodomain (YP_009724390.1, residues 1-1208) was codon-optimised for human expression, synthesised, and cloned into pcDNA.3.1(+) vector by GenScript. The construct was made with a “FUR 2P” set of stabilising mutations:^15^ R682S and R685S, which removed the polybasic cleavage site between S1 and S2; and K986P and V987P, which inhibited the propensity of the spike to assume the post-fusion conformation. The spike protein had a signal peptide derived from µ-phosphatase attached at the N terminus and a TEV-cleavage site, a foldon trimerisation motif, and a hexahistidine tag, all separated by short linkers, added at the C terminus.

The sequences coding for the variable regions of heavy (DQ168569.1) and light chains (DQ168570.1) of the CR3022 were added to those of the human constant regions to yield constructs coding for heavy and light chains of a Fab. The heavy chain construct had a human Ig heavy chain signal peptide at its N terminus for secretion and hexahistidine tag preceded by a TEV-cleavage site at its C terminus for purification; the light chain construct had a human Ig kappa chain signal peptide added at its N terminus. The constructs were synthesised with codons optimised for human expression by GenScript and cloned independently into pcDNA.3.1(+) vectors.

### Protein expression and purification

The proteins were expressed in suspension in Expi293F cells (Gibco) transfected with ExpiFectamine 293 (Gibco) at a density of 3 million cells per mL using either 1 mg of spike DNA or 0.5 mg of each heavy and light chain of the Fab per litre of culture. The cells were maintained in FreeStyle medium in a humidified, 8% CO_2_ atmosphere, at 37 °C, shaking at 125 rpm. The supernatants were harvested either twice, after 3–4 and 6–7 days, for the spike or once, after 6 days, for the CR3022 Fab.

The harvested, clarified supernatants were incubated overnight with TALON beads (Takara), washed briefly, eluted with imidazole, and loaded on a Superdex 200 Increase 10/300 GL column (GE Life Sciences) in a pH 7.4 phosphate buffer containing 70 mM NaCl. The Fab used in binding assays was then cleaved with recombinant TEV overnight and the protease, cleaved-off tags, and uncleaved proteins removed by incubation with TALON beads. The purity of all samples was confirmed with SDS-PAGE and the integrity of the spike trimers confirmed by cryoEM (described elsewhere^12^).

### Biolayer interferometry

Binding of CR3022 Fab to the SARS-CoV-2 spike proteins was measured in pH 7.4, 70 mM NaCl phosphate buffer with an Octet Red 96 (ForteBio) instrument. The spike glycoprotein at 20–40 µg/mL was immobilised for ~40 min on NiNTA sensors pre-equilibrated in the buffer. The association was then measured for 10 min followed by 30 min dissociation. Four independent experiments were used for the affinity calculation.

Association phases were analyzed as a single exponential function and plots of the observed rate (*k*_obs_) vs. CR3022 concentration gave the association and dissociation rate constants (*k*_on_ and *k*_off_) as the slope and intercept, respectively. The equilibrium dissociation constant (*K*_d_) was determined as *k*_off_/*k*_on_ and independently by analysis of the variation of maximum response with CR3022 concentration.

### Cryo-EM sample preparation and data collection

Prior to freezing, the Fab and S trimer were mixed at 3–1 molar ratio and incubated for 1 or 5 min, or mixed at 6–1 ratio and incubated for 40 h at 4 °C. The complexes obtained were then diluted to the final spike concentration of ~0.5 mg/mL and cryoEM samples prepared by applying 4 µL of a protein complex on a Quantifoil R2/2 grid of 200 mesh followed by a 4–4.5 s blotting with a Vitrobot MkIII and plunge freezing into liquid ethane.

Data were collected using EPU on a Thermo Scientific Titan Krios operating at 300 kV. Micrographs were collected using a Falcon 3 detector operating in electron-counting mode. Exposures were 60 s with a total dose of 33.6 e/Å^2^ fractionated into 30 frames, with a calibrated pixel size of 1.09 Å and a defocus range of 1.5–3 µm.

### Microscopy and data processing

Movies were aligned using MotionCor2^16^ and CTF-estimated using CTFFIND4^17^. Particles were picked using crYOLO^18^, with a manually trained model. Particles selected for all datasets were 2D classified using cryoSPARC^19^. Examples of 2D classes for 1 min, 5 min, and 40 h datasets are shown in Supplementary Fig. 2.

Particles contained in good 2D classes of the 40-h incubated complex (1.56 million) were chosen for structure solution and an ab initio model was calculated using cryoSPARC. These particles were 3D classified in RELION^20^ first into six classes. The best class was retained (288 k particles) and reclassified into further three classes. The resultant best class was chosen (88 k particles) and these particles were subjected to RELION Bayesian polishing^21^ and refined using cryoSPARC homogeneous refinement imposing C2 symmetry, coupled to CTF refinement, which generated a map at 3.9 Å resolution. In order to obtain higher resolution of the core regions of the complex, including the Fab fragments and RBD domains of S1, the particles were refined using cryoSPARC non-uniform refinement, generating a map at 3.7 Å resolution. A summary of the data processing workflow is shown in Supplementary Fig. 4. Maps had local resolution estimated using blocres^22^ implemented in cryoSPARC, followed by local resolution filtering and global sharpening^23^ in cryoSPARC.

### Model building

The map generated by non-uniform refinement was used to build a model of the core regions of the protein based on the pre-existing crystal structure of the CR3022 Fab bound to the RBD (PDB: 6W41^9^). This structure was built manually using COOT^24^ coupled to real-space refinement and validation using PHENIX^25^. The model obtained, built into the high-resolution density, was then used as the basis for the addition of less well-ordered regions of S1. The less ordered domains were added to the map and rigid body refined using PHENIX.

### Virus neutralisation assay

Confluent monolayers of Vero E6 cells (courtesy of National Institute for Biological Standards and Control, NIBSC) were incubated with ~14 PFU of SARS CoV-2 strain England/2/2020 (courtesy of Public Health England, PHE) and twofold serial dilutions of Fab over a concentration range of 50 μg/mL–1.53 ng/mL for 3 h at room temperature, in triplicate per condition. Inoculum was removed and cells were overlayed with virus growth medium containing avicell. Cells were incubated at 37 °C, 5% CO_2_. At 24 h post-infection, cells were fixed in 4% paraformaldeyhe and permeabilised with 0.2% Triton-X-100 and virus plaques were visualised by immunostaining using an anti-NSP8 antibody and action of HRP on a tetramethylbenzidine-based substrate as described previously for influenza viruses^26^. As a positive control for the assay, convalescent sera from COVID-19 infected patients were used.

### Reporting summary

Further information on research design is available in the Nature Research Reporting Summary linked to this article.

## Supplementary information

Supplementary Information

Reporting Summary

## Data Availability

Maps and models have been deposited in the Electron Microscopy Data Bank, http://www.ebi.ac.uk/pdbe/emdb/ (Accession numbers EMD-11647 and EMD-11648). Models have been deposited in the Protein Data Bank, https://www.ebi.ac.uk/pdbe/ (PDB ID codes 7A5S, and 7A5R). Source data are provided with this paper.

## Source data

Source Data
