# Supplementary material for: Antibody-mediated disruption of the SARS-CoV-2 spike glycoprotein
Source: Nat Commun. 2020 Oct 21;11:5337. doi: 10.1038/s41467-020-19146-5 (PMC7577971; doi:10.1038/s41467-020-19146-5)
Supplement: Supplementary file 1 — Supplementary Information [file 41467_2020_19146_MOESM1_ESM.pdf]

Supplementary Information

Antibody-mediated disruption of the SARS-CoV-2 spike glycoprotein

Wrobel, Benton, *et al*.

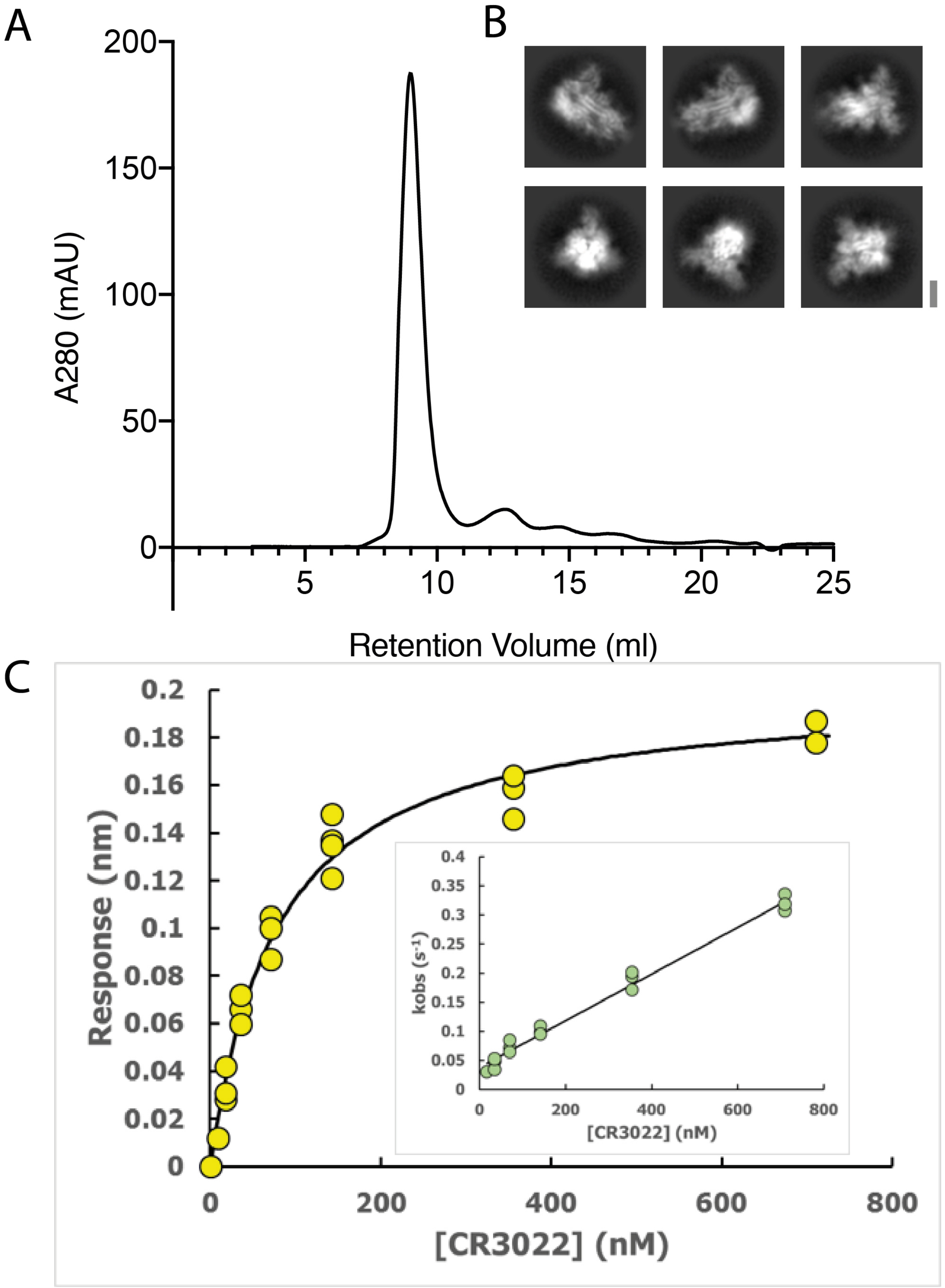

Supplementary Figure 1: Spike protein purification and biolayer interferometry. (**A**) Chromatogram from size exclusion chromatography showing the peak corresponding to the spike trimer used in this study around 9 mL of retention volume of S200 Increase column and (**B**) 2D averages from cryoEM dataset collected on this unbound trimer (the total of 171735 particles went into classification). All images are in the same scale, the scale bar is 5 Å tall. (**C**) Variation of maximum response with CR3022 concentration. Analysis gave a K_d of 78.8 ± 9.2 nM. Inset: Variation of k_{obs} with CR3022 concentration. Analysis gave k_{on} = 4 ± 1.3 x 10^5 $M^{-1}s^{-1}$ and k_{off} = 0.038 ± 0.006 s^{-1}, corresponding to a K_d calculated as k_{off}/k_{on} of 95.3 ± 15 nM.

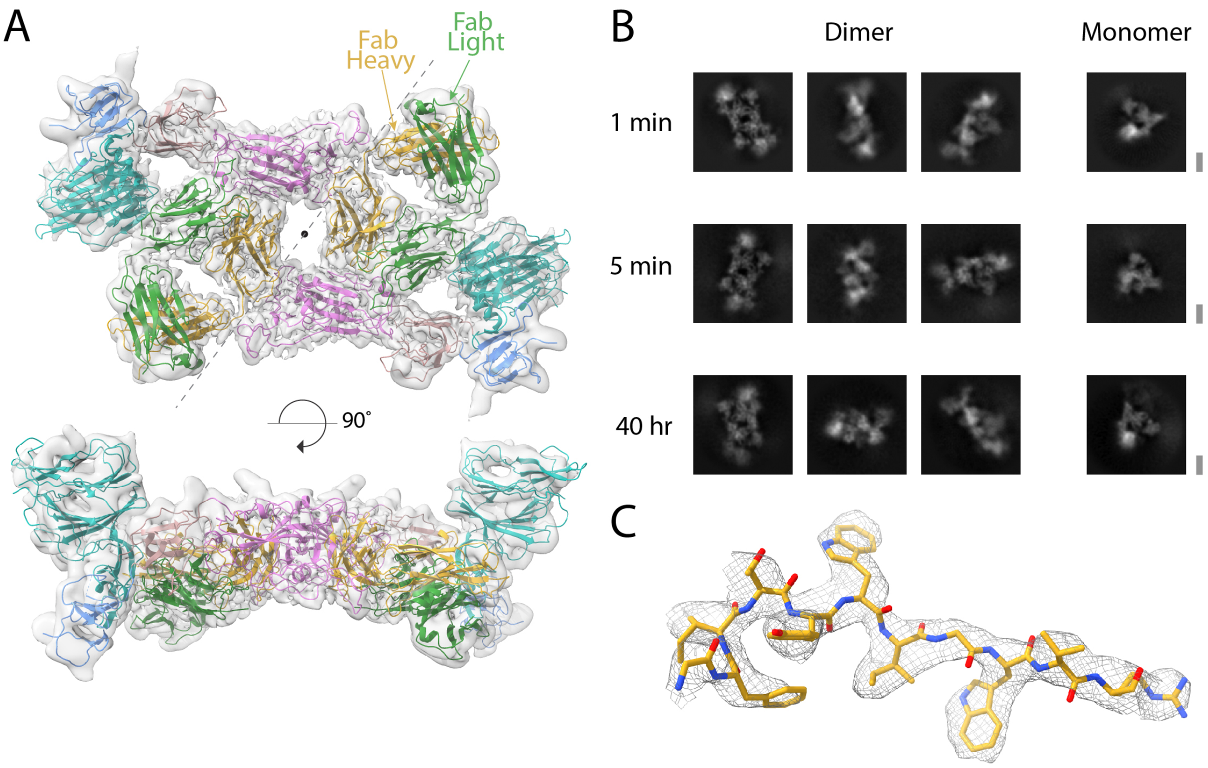

Supplementary Figure 2: CryoEM structure of complex formed by CR3022 Fab and SARS-CoV-2 spike. (**A**) CryoEM density (grey) and model of the dimer formed by CR3022 Fab and the S1 domain of SARS-CoV-2 spike shown in a ribbon representation and coloured as in Figure 1. (**B**) Representative 2D classes from different timepoints (with the total numbers of 192854, 163897, and 147198 particles going into classification for 1 min, 5 min, and 40 hr timepoints respectively) examined for the dimeric Fab-S1 complex (left) and a monomeric form (right) of the same complex. Images for each timepoint are in the same scale, the scale bars on the right are 5 Å tall. (**C**) Representative density (mesh) of the built model of the Fab heavy chain (yellow), residues 28-38.

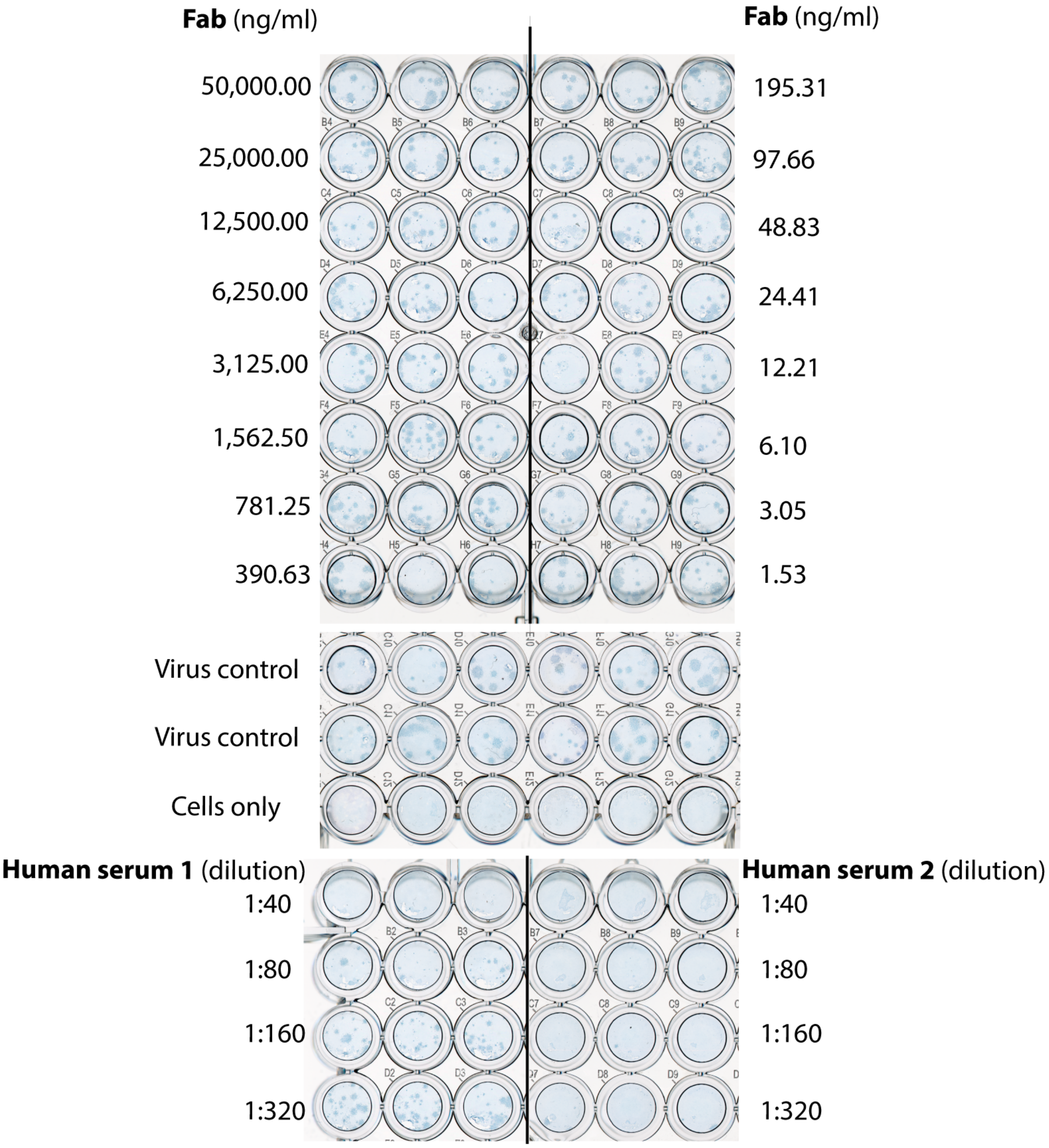

Supplementary Figure 3: SARS CoV-2 infection neutralisation by Fab. SARS CoV-2 infection neutralisation by Fab. Vero E6 cells were incubated for 3 hours with the SARS CoV-2 strain England/2/2020 in the presence or absence of Fab at a concentration range from 50 µg/ml-1.53 ng/ml or diluted human sera. The relevant sample was run in two-fold serial dilution series, as indicated, in triplicate (n=3). At 24 hours post-infection, cells were fixed, permeabilised and virus plaques were detected by immunostaining for viral protein NSP8.

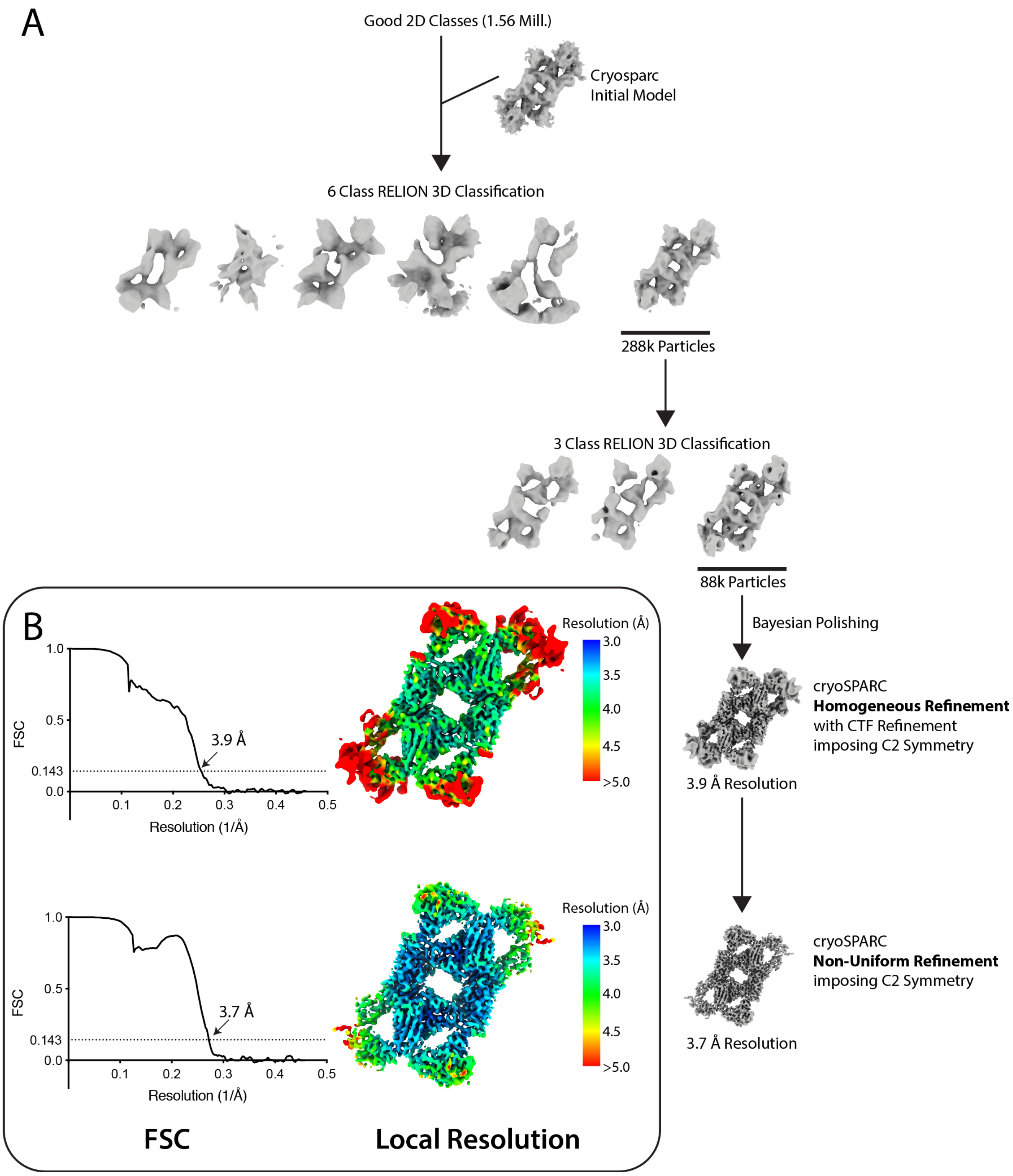

Supplementary Figure 4: cryoEM data processing workflow. (**A**) Classification scheme to obtain cryoEM maps. (**B**) Fourier Shell Correlation (FSC) curves and local resolution estimations of models.

Supplementary Table 1:

Cryo-EM data collection, refinement and validation statistics

	Homogeneous Refinement (EMD-11647) (PDB 7A5S)	Non-Uniform Refinement (EMD-11648) (PDB 7A5R)
**Data collection and processing**		
Voltage (kV)	300	300
Electron exposure (e–/Å^2)	33.6	33.6
Defocus range (μm)	-1.5 to -3.0	-1.5 to -3.0
Pixel size (Å)	1.09	1.09
Symmetry imposed	C2	C2
Final particle images (no.)	88 k	88 k
Map resolution (Å) FSC threshold = 0.143	3.9	3.7
Map resolution range (Å)	3.5-5.0	3.0-5.0
**Refinement**		
Initial model used (PDB code)	6W41	6W41
Model resolution (Å) FSC threshold = 0.5	4.1	3.8
Map sharpening *B* factor (Å^2)	-78.3	-71.3
Model composition		
Non-hydrogen atoms	16610	10608
Protein residues	2124	1358
Ligands	4	4
B factors (Å^2)		
Protein	46.8	51.3
Ligand	78.4	78.4
R.m.s. deviations		
Bond lengths (Å)	0.006	0.004
Bond angles (°)	0.942	0.832
Validation		
MolProbity score	1.77	1.64
Clashscore	5.63	3.78
Poor rotamers (%)	0.65	0.67
Ramachandran plot		
Favored (%)	92.67	92.26
Allowed (%)	6.95	7.59
Disallowed (%)	0.38	0.15
